# The Role of Methylation Modification in Neural Injury and Repair

**DOI:** 10.3390/ijms26115349

**Published:** 2025-06-02

**Authors:** Saizhen Lv, Yanyu Pan, Tiemei Zheng, Qianqian Cao, Bin Yu, Fengquan Zhou, Dong Wang

**Affiliations:** 1 Key Laboratory of Neuroregeneration of Jiangsu and Ministry of Education, Co-innovation Center of Neuroregeneration, Nantong University, Nantong 226001, China; lszfighting2022@163.com (S.L.); panyanyu010@163.com (Y.P.); 15110500711@163.com (T.Z.); qqcao@ntu.edu.cn (Q.C.); yubin@ntu.edu.cn (B.Y.); 2 Sir Run Run Shaw Hospital, Zhejiang University School of Medicine, Hangzhou 310016, China

**Keywords:** DNA methylation, histone methylation, RNA methylation, neural injury and repair, neural regeneration, epigenetic regulation

## Abstract

The diverse methylation modifications of DNA, histones and RNA have emerged as pivotal regulatory mechanisms of gene expression in multiple biological processes at the epigenetic level. They function by coordinating gene expression through impacting gene transcription, mRNA processing and maturation, protein translation and metabolism. Changes in methylation profiles of nucleic acids and histones have been observed in many different types neural injuries in both the central nervous system and the peripheral nervous system, such as 5-methylcytosine in DNA, N6-methyladenosine in RNA and methylation of lysine residues in various histones. Importantly, altering these modifications plays key roles in regulation of neural injury and repair. In this review, we highlight recent research advances of the methylation-related epigenetic modifications in multiple aspects of neural injury and regeneration, including neural protection, axon regeneration, microenvironment modulation and neural functional recovery. We also discuss the current unsolved problems in the field and propose potential future research directions.

## 1. Introduction

Insight into the molecular mechanisms of spatiotemporal gene expression regulation is critical for nerve injury repair. This includes studies on both central (e.g., brain, spinal cord and optic nerve) and peripheral (e.g., sciatic nerve) nerve injury [1,2,3,4]. Many previous studies have focused on examining the effect of transcription factors [5,6], kinases [7,8] or the single functional proteins in nerve injury repair [9,10,11], whereas recent increasing reports have indicated the vital roles of epigenetic regulation in this process, including the methylation modifications of histones, DNA or RNA [12,13,14,15]. During nerve injury and repair, similar epigenetic regulations of gene transcription have been demonstrated in many studies as basic gene expression modulation mechanisms.

Methylation is the most common epigenetic modification in diverse biological processes. Methylation modification is achieved by the catalytic transfer of methyl groups from an active methyl compound, such as S-adenosylmethionine, to other receptors [16,17]. It can occur in DNA, histones and RNA, all of which require their respective writers (methyltransferases), erasers (demethylases) and readers (binding proteins) [18,19,20,21]. The dysregulation of methylation levels often occurs during nerve injury, degeneration and regeneration. 5-methylcytosine (5mC), the most common DNA methylation modification, and its hydroxymethylation products 5-hydroxymethylcytosine (5hmC), have been reported to be involved in the regulation of nerve injury and regeneration [17,22]. In addition, another form of DNA methylation, N6-methyldeoxyadenosine (6mA), was also observed during the neurite outgrowth [23]. For histone methylation, trimethylation of histone 3 lysine 9 (H3K9me3) or histone 3 lysine 27 (H3K27me3) has been reported following axotomy [24,25,26]. Moreover, recent studies have revealed that RNA N6-methyladenosine (m^6^A) exhibits novel regulatory functions in modulating nerve injury and regeneration [27,28,29].

In the present review, we summarize recent studies involved in the role of altered methylation levels in DNA and histones or RNA during nerve injury and regeneration, highlighting some discoveries with fascinating insights into the effect of methylation on nerve repair. We also discuss some potential issues facing the field and suggest potential future research directions.

## 2. Overview of Methylation in DNA, Histones and RNA

### 2.1. DNA Methylation

DNA methylation serves as a mark for repressing gene expression by influencing the binding of regulatory factors and altering chromatin structure. DNA methylation in 5mC, one of the most extensively characterized epigenetic modifications, plays pivotal roles in transcriptional regulation. 5mC dysregulation has been linked to processes such as genomic imprinting, X chromosome inactivation and retrotransposon silencing [30]. 5mC regulates gene expression via direct and indirect manners. The 5mC present in the promoter region directly obstructs transcription factor (TF) binding by cytosine methylation within its motif. The indirect manner for gene repression is via methyl-CpG-binding domain (MBD) proteins that recognize methylated CpGs and recruit histone deacetylases to methylated DNA to contribute to the transcriptional repression [31,32].

DNA methylation is mediated by DNA methyltransferases and demethylases. The establishment and maintenance of 5mC are carried out by two families of DNA methyltransferases: the ‘de novo’ methyltransferases, DNA methyltransferase 3 (*Dnmt3*) family (*Dnmt3a* and *Dnmt3b*) and the ‘maintenance’ methyltransferase DNA methyltransferase 1 (*Dnmt1*) [33]. DNA demethylation occurs via two major pathways. The first is known as “passive” or replication-dependent DNA demethylation. The DNMT1–ubiquitin-like with plant homeodomain (PHD) and ring finger domains 1 (UHRF1) maintenance methyltransferase complex binds to hemi-methylated 5mC sequences and restores symmetrical 5mC on the newly synthesized strand during DNA replication. However, Tet methylcytosine dioxygenase (TET) enzymes catalyze the oxidation of 5mC to 5hmC, which is poorly recognized by the DNMT1-UHRF complex, and these DNA modifications are thereby diluted during successive cycles of DNA replication. The second is known as “active” or replication-independent DNA demethylation. The TET family of proteins (TET1, TET2 and TET3) can oxidize the methyl group on 5mC, converting it into 5hmC. TET proteins also catalyze further oxidation of 5hmC to form 5-formylcytosine (5fC) and 5-carboxylcytosine (5caC) [34,35]. Additionally, activation-induced deaminase/apolipoprotein B mRNA-editing catalytic polypeptide-like (AID/APOBEC) can deaminate 5mC and 5hmC, resulting in thymine (Thy) and 5-hydroxymethyl-uracil (5hmU) [36]. Finally, the products of these pathways are recognized and excised by the base excision repair machinery, mediated by thymine DNA glycosylase (TDG) and/or single-stranded monofunctional uracil DNA glycosylase (SMUG1), and replaced by unmethylated cytosine [18,37].

The functions of DNA 5mC and its demethylated derivatives depend on several binding proteins, often referred to as “readers”. Previous studies have identified several structurally distinct domain proteins that bind to 5mC, including the methyl-CpG-binding domain (MBD) family proteins (e.g., MBD1, MBD2, MBD3 and methyl-CpG-binding protein 2, MeCP2), SRA-domain proteins (e.g., UHRF1 and UHRF2) and specific subsets of zinc finger proteins (Zinc finger and BTB domain-containing 33, ZBTB33 and ZFP57 zinc finger protein, ZFP57) and a few other transcription factors (Kruppel-like factor 4, KLF4) [38,39,40]. In addition to the above 5mC binding proteins, the role of the CCCTC-binding factor (CTCF) is to mediate 5mC methylation-sensitive binding with DNA located genome-wide by functioning as a transcriptional insulator, repressor or activator [41,42]. Pioneering studies have highlighted the specificity of various ‘readers’ for different oxidized cytosine bases, like the RING finger associated (SRA) domain of UHRF2 for 5hmC and zinc finger proteins Spalt-like transcription factor 4 (SALL4) for 5hmC [34,43,44].

In addition to 5mC sites, DNA methylation at 6mA sites is another prevalent DNA modification [45,46]. Recent reports indicated that the potential effect of 6mA modification was involved in the regulation of DNA structure and gene expression, especially that 6mA was associated with the repression and silencing of genes in mouse embryonic stem cells during development [47,48]. In contrast, compared with the repressive gene expression function of 5mC in DNA, the potential function of 6mA modification in mammals remains an active area of ongoing research. The N-6 Adenine-Specific DNA Methyltransferase 1 (N6AMT1) complex, the METTL3-METTL14 complex and METTL4 have been identified as ‘writers’ proteins [49,50,51]. The demethylase AlkB homolog 1, histone H2A dioxygenase (ALKBH1), has been recognized as the ‘eraser’ protein for 6mA [52], while YTH N6-methyladenosine RNA binding protein C1 (YTHDC1) has been validated as the ‘reader’ protein for 6mA in mammals [53]. The functions of DNA methylation and its derivative products likely depend on recognition by cell-specific binding proteins, although this process is being actively explored.

### 2.2. Histone Methylation Modification

Histone methylation predominantly occurs on lysine (K) residues of histones H3 and H4, existing in three distinct states: mono- (me1), di- (me2) and trimethylation (me3). For arginine (R) methylation, two major forms are observed: omega-NG-monomethyl-Arg (MMA) and asymmetric dimethyl-Arg (ADMA). These modifications predominantly localize to specific histone sites, including H3R2, H3R17, H3R26 and H4R3. Histone Lys methylation serves as a regulatory mark for both gene activation and repression and is involved in regulating the cell cycle, genome stability and nuclear architecture [19,54], while the association of histone Arg methylation marks with gene expression is a direction for further studies [55].

The methylation of specific lysine residues, such as Lys4 on histones H3 (H3K4), H3K36 and H3K79, is commonly associated with gene activation, while H3K9, H3K27 and H4K20 are typically regarded as repression marks [54]. The distinct methylation states (me1/me2/me3) of lysine residues in histone H3 exhibit characteristic genomic distributions and functional associations. H3K4me1 is predominantly localized to enhancer regions, while H3K4me2 marks transcriptional initiation sites at the 5′ ends of transcribed genes, and H3K4me3 shows a canonical enrichment pattern at promoters of actively transcribed genes [56,57,58]. Regarding H3K36 methylation, H3K36me2 displays a broad distribution across both genic and intergenic regions, whereas H3K36me3 is predominantly enriched within gene bodies [59]. H3K79 methylation demonstrates enrichment in coding regions and correlates with active transcription [60].

In the case of H3K9 methylation, H3K9me2 and H3K9me3 are primarily associated with transcriptional repression and heterochromatin formation [61,62]. In contrast, H3K9me1 is detected near transcription start sites (TSSs) of actively transcribed genes [63]. For H3K27 methylation states: H3K27me1 localizes within gene bodies of transcriptionally active genes; H3K27me2 occupies inter- and intragenic regions, potentially serving to suppress inappropriate promoter or enhancer activation; and H3K27me3 shows strong enrichment at PRC2-bound genomic regions, with additional low-level distribution across other chromosomal areas [64]. H4K20me1 marks coding regions of minimally transcribed genes and accumulates in parental nucleosomes during cell division [65]. The stepwise methylation from H4K20me1 to H4K20me2/me3 plays critical roles in genome stability maintenance, DNA damage response pathways, chromatin compaction processes, DNA replication regulation and nucleosome turnover dynamics [66].

Lysine methylation in histones is regulated by methyltransferases (KMTs), which possess the catalytic SET domain, such as euchromatic histone lysine methyltransferase 1 (*Ehmt1*) and *Ehmt2*, which generate H3K9me1/2 [67,68], enhancer of zeste 2 polycomb repressive complex 2 subunit (*Ezh2*) for H3K27me2/3 [64,69], SUV39H1 histone lysine methyltransferase (*Suv39h1*) and *Suv39h2* for H3K9me2/3 [70,71] and the lysine methyltransferase 2 (KMT2) family for H3K4me2/3 [72]. The removal of histone methylation is catalyzed by demethylases (KDMs) containing the jumonji-C domain, such as *Kdm6a* or *Kdm6b*, which demethylate H3K27me2/3 [73,74]. Previous studies indicated that H3K4me3 is enriched at TSSs and promotes transcription through the recruitment of PHD-domain-containing proteins involved in transcription initiation, such as TATA-box-binding protein associated factor 3 (TAF3) [58]. Moreover, recent reports reveal that H3K4me3 is also essential for the eviction of paused RNAPII and transcriptional elongation during transcription [75]. It is important to note that numerous studies have highlighted a highly interconnected relationship between DNA methylation and histone lysine methylation [76,77]. Studies have shown that DNMT3L recognizes unmethylated H3K4 tails to facilitate de novo DNA methylation through recruiting or activating DNMT3A2. Consequently, methylation of H3K4 may counteract DNA methylation [78]. Furthermore, the binding and activity of DNMT3A co-localize with H3K36me2 in non-coding regions of euchromatin. H3K36me2 is essential for recruiting DNMT3A and maintaining DNA methylation at intergenic regions, indicating that histone methylation directly modulates DNA methylation patterns [59]. The modification state and sequence of DNA can influence the methylation state of associated histones in chromatin, while the histone lysine methylation state can, in turn, affect the modification of DNA itself.

### 2.3. RNA Methylation Modification

RNA methylation is a widely recognized form of post-transcriptional regulation found in mRNA, tRNA, rRNA, snRNA and snoRNA. It plays a pivotal role in various biological processes [79,80,81]. Currently, the processes of m^6^A, 5-methylcytosine (m5C) and N1-methyladenosine (m1A) modification in RNAs are well understood. These modifications are crucial regulators of gene expression during development and disease [82,83]. The m^6^A modification is also coordinated by a set of “writers,” “erasers” and “readers” proteins. Among “writer” proteins, the first identified m⁶A methyltransferase is the *Mettl3* catalytic subunit, which forms a multiprotein complex with accessory subunits like *Mettl14* and WT1-associated protein (*Wtap*) [84]. The second identified m⁶A methyltransferase, *Mettl16*, targets substrates including U6 snRNA and the human methionine adenosyltransferase 2A (MAT2A) mRNA [85,86]. The m^6^A modification is removed by two eraser enzymes, the fat mass- and obesity-associated proteins (*Fto*) and *Alkbh5* [87,88]. The biological function of m^6^A is mediated by m^6^A-recognizing proteins (readers), such as the YTH domain family proteins (*Ythdf1*, *Ythdf2* and *Ythdf3*), the insulin-like growth factor 2 mRNA binding protein family proteins (*Igf2bp1*, *Igf2bp2* and *Igf2bp3*) and *Ythdc1* and *Ythdc2*. YTHDFs are primarily localized in the cytoplasm, YTHDC1 are nuclear readers, and IGF2BPs are enriched across the 3′ UTR of RNA [89,90].

In addition to m^6^A, recent studies have highlighted the vital role of m5C in RNA. The RNA methyltransferase NOP2/Sun RNA Methyltransferase 2 (*Nsun2*), which is involved in m5C modification, has been identified through whole-genome transcription mapping [91,92,93]. Interestingly, the m^6^A-binding protein YTHDF2 has also been found to directly bind m5C in RNA, which plays a role in pre-rRNA processing [94]. Meanwhile, m1A modification is less frequent in mRNA, with the exception of the mitochondrial-encoded gene *Nd5* [95,96]. Additionally, m^6^A and other RNA methylation modifications are also present in long non-coding RNAs (lncRNAs) and microRNAs (miRNAs) [97]. WTAP mediates the alteration of m^6^A levels in lncRNA NORAD, which impacts NORAD decay through YTHDF2 [98]. Furthermore, METTL3 increases m^6^A levels in pri-miR-1246, thereby promoting its maturation into miR-1246 [99]. The dynamic deposition of RNA methylation modifications regulates mRNA metabolism by reader proteins through impacting RNA nuclear export, alternative splicing, decay and translation [100,101]. Specifically, YTHDF1 binds to m^6^A sites in target mRNAs to facilitate ribosome loading and accelerate translation [102]. Conversely, YTHDF3 not only assists YTHDF1 in enhancing translation efficiency but also facilitates YTHDF2-mediated mRNA decay [103]. Nuclear-localized YTHDC1 governs the splicing and nuclear export of m^6^A-modified mRNAs, while the cytoplasmic reader YTHDC2 enhances target transcript translation while simultaneously reducing their stability [104,105,106]. IGF2BPs modulate the stability of m^6^A-containing transcripts by recruiting RNA stabilizers and promoting mRNA translation, likely through recruitment of eukaryotic translation initiation factor (eIF) proteins [107,108]. Regarding RNA m5C modification, NSUN2 decreases *Pten* mRNA expression by regulating alternative splicing of target mRNAs in an m5C-dependent manner [109].

## 3. DNA Methylation Changes Following Nerve Injury

**Figure 1:**
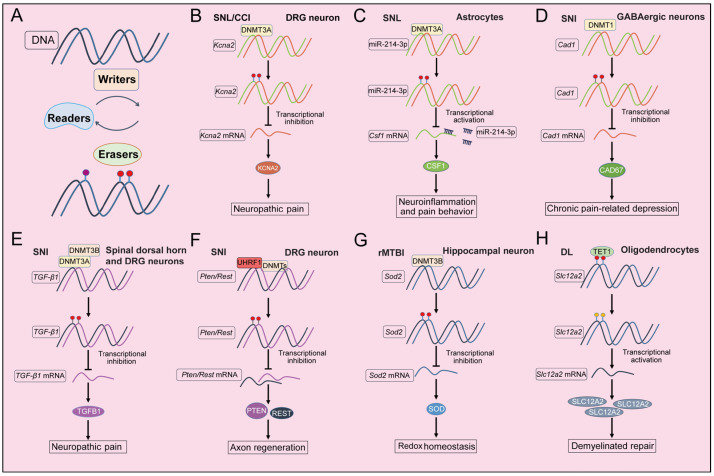
Mechanism of DNA methylation regulating nerve regeneration and repair in different models of nerve injury. The mechanism of DNA methylation in neuronal cell following nerve injury. (**A**) The schematic diagram of DNA methylation is modulated by Writers (methyltransferases, e.g., DNMT3A, DNMT3B and DNMT1), Erasers (proteins involved in the DNA demethylation process, e.g., TET1, TET2 and TET3) and Readers (binding proteins for DNA 5mC and its demethylated derivatives, e.g., MBD1 and UHRF1) proteins. (**B**–**E**) DNA methylation regulates the function of DRG, astrocytes and lumber spinal cord neurons in pain following nerve injury. (**F**) DNA methylation regulates the axon regeneration in DRG neurons following nerve injury. (**G**,**H**) DNA methylation regulates redox homeostasis or demyelinated repair in the hippocampal neuron or oligodendrocyte following nerve injury. SNL (Spinal nerve ligation); CCI (Chronic constriction injury); DRG (Dorsal root ganglia); SNI (Sciatic nerve injury); rMTBI (repeated mild traumatic brain injury); DL (Demyelinated lesions).

Recent studies have increasingly focused on the role of DNA methylation in nerve injury, coinciding with advancements in methylation detection methods. Table 1 provides a summary of these studies, including details on animal species, nerve injury models, experimental timelines, methylation detection techniques, sample types and the specific types of DNA methylation examined. Figure 1 illustrates the regulatory mechanisms of DNA methylation in key processes following nerve injury, including pain, axon regeneration, redox homeostasis and remyelination.

### 3.1. Brain Trauma

Traumatic brain injury (TBI), a common neurological trauma, triggers dynamic DNA methylation alterations in the brain. Emerging evidence suggests these epigenetic modifications critically influence both injury and repair. In blast-related TBI, previous reports indicated that DNA methylation perturbations occur in neurons and glial cells sorted from whole brain tissue and identified that decreased expression of the aralkylamine N-acetyltransferase (*Aanat*) gene correlated with increased methylation levels in blast samples [110]. Another study found that elevated levels of DNA methylation of gene encoding brain-derived neurotrophic factor (*BDNF*) in DNA extracted from cerebrospinal fluid may be associated with better recovery following severe TBI in adults. Furthermore, *BDNF* DNA methylation may serve as an early post-injury biomarker, helping to explain the heterogeneity of outcomes after TBI [111]. These observations suggest a dynamic interaction between DNA methylation levels and the progression of TBI, which could be used as a prognostic factor for TBI outcomes.

Interestingly, beyond global changes in methylation, distinct DNA methylation levels have been reported in specific neuron cell types following brain injury [112]. A subpopulation of activated microglia/macrophages with hypomethylation was detected in the brain at the early stage of TBI [112]. Moreover, hypermethylation was also observed in the highly reactive astrocytes following TBI [113]. Additionally, injury-induced relocalization of DNMT1 was examined in reactive astrocytes during secondary injury following brain trauma [114]. Thus, evidence suggests that injury-induced epigenetic reprogramming exhibits cell type-specific patterns in distinct brain cell populations. Neurons, microglia/macrophages and astrocytes demonstrate differential methylation states that correlate with their specialized functional responses post-injury.

At the mechanistic level, DNA methylation has been shown to affect mitochondrial biogenesis by regulating mitochondrial transcription factor A (*Tfam*) in the hippocampus of rats with repeated mild traumatic brain injury (rMTBI) [115]. DNMT3B-mediated DNA methylation plays a critical role in redox homeostasis by regulating superoxide dismutase 2 (*Sod2*) expression in the hippocampus following rMTBI [116]. Additionally, in lysolecithin-induced demyelinating injury, fewer remyelinated axons were detected in the lesions of demethylase-deficient *Tet1*-iKO (*Tet1* flox/flox; NG2-CreER; R26-EYFP) mice compared to controls at 28 days post-lesion. TET1-mediated demethylation of the target gene, inositol 1,4,5-trisphosphate receptor type 2 (*Itpr2*), is required for myelination and remyelination [117]. Thus, altered DNA methylation appears to be a common response in TBI. It seems that, compared with changes in RNA metabolism and protein function, the altered DNA methylation modifications in TBI do not induce deep consequences.

### 3.2. Spinal Cord Injury

Spinal cord injury (SCI) has been a significant clinical challenge for centuries, primarily due to the loss of axon regeneration and neuronal dysfunction. Previous studies have also identified common differentially methylated genes between SCI and peripheral nerve injury (PNI). Some of these genes (arylsulfatase B, *Arsb* and Reln) are involved in the regulation of axon regeneration [118]. Distinct dynamic 5hmC modification patterns were observed in the dorsal root ganglia (DRG) after T8 dorsal column transection and PNI. These modifications were associated with changes in several regeneration-associated genes (RAGs), including activating transcription factor 3 (*Atf3*), *Bdnf* and SMAD family member 1 (*Smad1*) [119]. Moreover, altered Bdnf methylation levels in lumbar spinal tissue were also examined in a neonatal spinal transection model [120]. Interestingly, recent reports have shown that decreased DNA methylation levels (including non-CpG methylation region-associated genes) were functionally enriched in neuronal synaptic connection creation and axon regeneration following SCI [121]. These findings suggest that compared to PNI, SCI triggers spatiotemporally specific patterns of DNA methylation and demethylation processes. Furthermore, it is critical to consider the stage-dependent functional divergence of key enzymes involved in methylation dynamics across different phases of injury progression.

It has been reported that folate induces axonal regeneration after rodent SCI and optic nerve injury by restoring DNA methylation in a folate receptor 1 (*Folr1*)-dependent manner [122]. Moreover, this enhanced axonal regeneration phenotype was found to be inherited transgenerationally, even without folic acid supplementation in subsequent generations [123]. Interestingly, a recent study observed that both 5hmC and 5mC levels at enhancer sites are prevalent and correlated with transcriptional changes during axon regeneration. Distinct roles of 5hmC and 5mC at transcription factor binding sites were also identified in the folate-induced transgenerational regeneration phenotype following SCI [124]. Additionally, 5hmC levels within the brain increased following SCI, especially during the acute and subacute stages. Correspondingly, the mRNA levels of TET family genes (*Tet1*, *Tet2* and *Tet3*) were also elevated. Ascorbic acid administration and exercise training promoted axonal sprouting and regeneration within the lesion cavity of the spinal cord by enhancing 5hmC levels and TET gene expression [125,126]. Modulating 5hmC dynamics could, therefore, offer a promising strategy to improve SCI outcomes.

Moreover, DNA hydroxymethylation and TET1 are essential for adult myelin repair and regulation of the axonal–myelin interface by targeting solute carrier family 12 member 2 (*Slc12a2*) in adult oligodendrocytes during the repair of demyelinated lesions (DL) [127]. Therefore, targeting dynamic DNA methylation levels holds significant therapeutic potential for promoting SCI repair, and the precise molecular mechanisms underlying methylation-mediated neural regeneration should be characterized in future studies.

### 3.3. Optic Nerve Injury

In addition to spinal cord injury, optic nerve injury (ONI) is another prevalent condition affecting the CNS. The failure of axon regeneration and the death of retinal ganglion cells (RGCs) lead to severe visual dysfunction following ONI. Despite its significance, only a few studies have explored the role of DNA methylation in ONI.

One study demonstrated that restoring youthful DNA methylation patterns and transcriptomes by ectopically expressing the Oct4, Sox2 and Klf4 genes (OSK) in mouse RGCs could promote axon regeneration following ONI. This approach also reversed vision loss in a mouse model of glaucoma and in aged mice, with the process requiring the DNA demethylases TET1 and TET2 [128]. However, whether modulating DNA methylation levels or altering the expression of methylation-related proteins can enhance optic nerve repair following ONI remains unclear. Another study indicated that DNA methylation-mediated regulation of Na+/K+-ATPase supports axonal regeneration in both purified embryonic and adult retinal neurons in mice [129].

### 3.4. Sciatic Nerve Injury

The mature central nervous system (CNS) lacks intrinsic regenerative capacity. CNS injuries trigger a prolonged inflammatory response involving microglia/macrophages and scar formation by astrocytes/fibroblasts, which create a hostile environment that blocks axon regeneration [130,131]. In contrast, peripheral nerves maintain a limited capacity for axon regeneration following injury. The sciatic nerve injury model is a classic system for studying PNI. Axon regeneration in this context depends on both the intrinsic transcription of RAGs, such as *Atf3* [132] and dual leucine zipper kinase (*Dlk*) [7], and a permissive microenvironment. Critically, Schwann cells facilitate regeneration through phagocytosis of myelin debris and secretion of neurotrophic factors [133,134]. These distinct mechanisms induce the disparate regeneration abilities of the CNS and PNS after injury. While recent studies have enhanced our understanding of DNA methylation’s role in sciatic nerve injury, its precise function in axon regeneration remains to be fully validated.

Different mechanisms have been proposed for the role of DNA methylation in nerve repair following sciatic nerve injury (SNI). The increased 5hmC levels and upregulated TET3 expression in DRG neurons have been shown to support axon regeneration after PNI [119]. Meanwhile, DNA demethylation-induced transcriptional hub gene *c-Myc* promotes axon regeneration by driving the upregulation of RAGs post-PNI [135]. Conversely, perturbing DNA methylome changes by pharmacological inhibition or activation of DNA methylation markedly attenuated the axon growth capacity of the preconditioned DRG neurons [136]. In addition, studies indicated that UHRF1 promotes robust axon regeneration by targeting DNA methylation at promoter regions of inhibitory genes, including phosphatase and tensin homolog (*Pten*), cyclin dependent kinase inhibitor 1a (*Cdkn1a*) and RE1 silencing transcription factor (*Rest*) [137]. In addition, a recent study identified another DNA methylation modification, 6mA, whose primary function is to condense chromatin and repress gene expression. 6mA was found to play a significant role in axon regeneration, and knockdown of its demethylase ALKBH1 impaired axon regeneration following sciatic nerve injury [23].

In addition to axon regeneration, the proliferation, migration and myelination of Schwann cells are crucial for peripheral nerve injury and repair. Recent studies have shown that alterations in CpG methylation following injury are limited to specific regions and are often confined to injury sites. Significant changes in DNA methylation occur near TF-binding motifs of *c-Jun* and other AP-1 family members, which are expressed as part of the Schwann cell phenotype during repair [138]. On the other hand, restoring global DNA methylation in these mice was shown to improve Schwann cell myelination [139]. Additionally, DNMT1 was found to inhibit the proliferation and myelination of Schwann cells by mediating RUNX family transcription factor 3 (*Runx3*) via the JAK/STAT signaling pathway [140]. The regulation of transcription factors expression through DNA methylation constitutes a critical epigenetic mechanism by which Schwann cell functional plasticity is dynamically controlled.

Spinal nerve ligation (SNL) is another commonly used surgical model for studying neuropathy and allodynia in PNI. PNI leads to significant remodeling of the methylome in the DRG [141]. Alterations in the expression of DNA methylation and hydroxymethylation enzymes (TET1-3) have been observed in the brain of a rat model of neuropathic pain [142]. A recent report indicated that SNL induces consistent low-level hypomethylation at CpG sites in the DRG during both the acute and chronic phases of neuropathic pain. This methylation reprogramming is correlated with increased gene expression variability. Methyl donor deficiency or inhibition of DNA methyltransferases leads to hypomethylation, causing long-lasting pain hypersensitivity. Thus, restoring DNA methylation has been proposed as a potential therapeutic strategy to alleviate neuropathic pain [143]. For instance, intraperitoneal injection of 5-azacytidine has been shown to reduce nerve injury-induced pain in rats by modulating DNA methylation [144]. SNL has also been shown to induce demethylation of the promoter region of the C-X-C motif chemokine receptor 3 (*Cxcr3*) gene and decrease the expression of DNMT3B [145]. Additionally, DNMT3A is predominantly expressed in postsynaptic neurons of the ipsilateral nucleus accumbens in neuropathic pain induced by peripheral nerve injury [146]. In injured DRG neurons, DNMT3A affects neuropathic pain by repressing the expression of potassium voltage-gated channel subfamily a member 2 (*Kcna2*) via the activation of octamer transcription factor 1 (OCT1) [147].

Interestingly, a recent report highlighted that SNL induced DNMT3A-regulated hypermethylation of the miR-214-3p promoter, modulating colony stimulating factor 1 (CSF1) production in astrocytes during SNL-induced neuroinflammation and pain behavior [148]. And the reader protein, MeCP2, modulates transcriptional programs in the superficial dorsal horn and DRG, contributing to the persistence of pathological pain [149]. Moreover, DNMT3A and DNMT3B have been shown to induce hypermethylation at the promoter region of the antiallodynic cytokine transforming growth factor beta 1 (*Tgfβ1*) in the spinal dorsal horn in neuropathic pain [150]. Another study revealed that DNMT1 mediates chronic pain-related depression by promoting methylation at the CpG-rich glutamate decarboxylase 1 (*Gad1*) promoter and downregulating *Gad67* expression, which affects GABAergic neuronal activation in the central amygdala [151].

In addition to the peripheral nerve ligation (PNL) model, the chronic constriction injury (CCI)-induced neuropathic pain model is also widely used in pain research. A previous study found that decreased expression of GAD67 may be associated with increased methylation of the *Gad1* promoter, which could be regulated by elevated levels of DNMT3A, DNMT3B and MeCP2, along with decreased expression of MBD2 in the lumbar spinal cord of the CCI model [152]. Furthermore, treatment with 5-azacytidine, a DNA methyltransferase inhibitor, has been shown to alleviate neuropathic pain in CCI rats by significantly reducing global DNA methylation levels and MeCP2 expression in the spinal cord [153]. Lastly, increased binding of MeCP2 and HDAC1 at the *Oprm1* promoter sites negatively regulates MOR expression in injured DRG, reducing the efficacy of opioid analgesia in CCI model [154].

This evidence highlights DNA methylation as a central epigenetic modification that coordinates both peripheral nerve repair and neuropathic pain through distinct mechanisms. Specifically, DNMTs and TETs enzymes mediate dynamic modifications at enhancer and promoter regions, regulating neuronal hyperexcitability and glial cell activation in nerve injury-induced neuropathic pain.

**Table 1:** List of articles on DNA methylation in nerve injury and regeneration.

Animal	Injury Model	Timeline	Methods	Methylation Type	Sample	Ref.
Mice	Sciatic nerve crush	0, 3 days	Dot blot Assay	6mA	DRG	[23]
Rat	T10–T11 spinal cord transection/ Bilateral sciatic nerves ligation	0, 14 days	WGBS/MeDIP-Seq	5mC/5hmC	Spinal cord/Sciatic nerve	[118]
Mice	T8 spinal cord transection	24 h	5hmC enriched ChIP-Seq,	5hmC	L4–L6 DRGs	[119]
Rat	T8–T10 spinal cord transection	Postnatal day 10	Bisulfite sequencing PCR (BSP)	5mC/5hmC	Spinal cord	[120]
Rat	C3 dorsal column spinal cord injury	N/A	5hmC enriched ChIP-Seq, WGBS	5mC/5hmC	Spinal cord	[124]
Mice	lysolecithin-induced demyelinating injury	N/A	RRHP	5hmC	OPCs/OLs	[127]
Mice/rat	T9 dorsal hemisection	0, 2 h, 0.5 h, 1 day, 7 days	Bisulfite sequencing	5mC/5hmC	L4-L6 DRG	[135]
Rat	Sciatic nerve transection	0, 1 day, 7 days	MeDIP-Seq	5mC	L4–L6 DRGs	[136]
Mice	Sciatic nerve transection	0, 7 days	WGBS	5mC/5hmC	Sciatic nerve	[138]
Mice	Diabetes-induced myelin sheaths neuropathy	6 months	RRBS	5mC/5hmC	Sciatic nerve	[139]
Rat	Spinal nerve ligation	0, 24 h	RRBS	5mC/5hmC	L5 DRG	[141]
Rat	T9 moderate contusion injury	0, 1 h, 1 week, 1 month, 3 months	DNA Dot blot Assay	5mC/5hmC	Brain motor cortex	[125]
Rat	T9 moderate contusion injury	0, 12 weeks	DNA Dot blot Assay	5mC/5hmC	Brain motor cortex	[126]
Rat	C3 dorsal column spinal cord injury	N/A	MeDIP microarrays	5mC/5hmC	Spinal cord	[155]
Mice	T10 dorsal hemisection	0,1 day,3 days, 7 days	MeDIP-chip	5mC	L4–L6 DRGs	[156]
Rat	Spinal nerve ligation	0, 3 days, 3 weeks	DREAM/RRBS	5mC/5hmC	Brain, spinal cord, and L5-6 DRG	[143]

Note: WGBS (Whole-genome bisulfite sequencing); RRHP (Reduced representation hydroxymethylation profiling); MeDIP-Seq (Methylated DNA immunoprecipitation sequencing); DREAM (Digital restriction enzyme analysis of methylation); RRBS (Reduced representation bisulfite sequencing); OPCs (Oligodendrocyte precursor cells); OLs (Oligodendrocytes); DRG (Dorsal root ganglia).

## 4. The Effect of Histone Methylation in Nerve Injury

**Figure 2:**
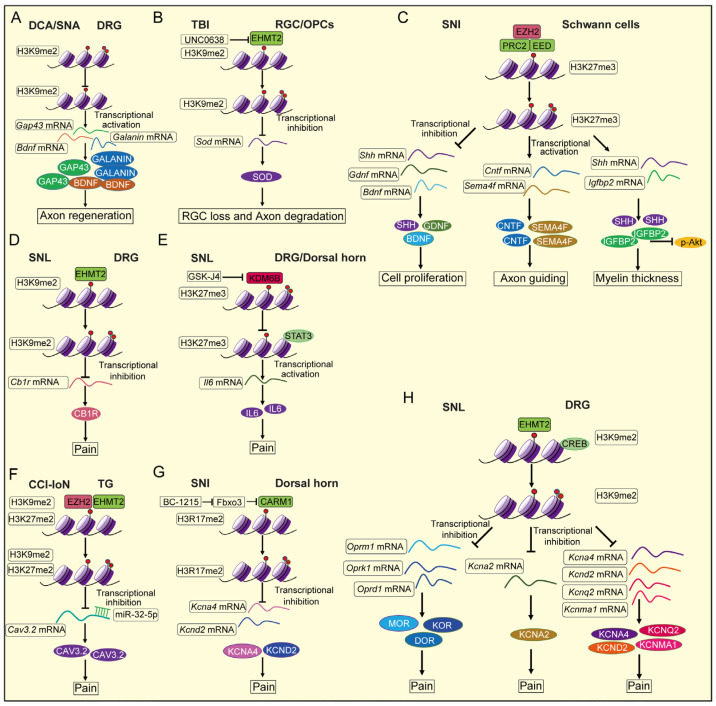
The mechanism of histone methylation in neuronal cell following nerve injury. (**A**) Histone methylation regulates the axon regeneration in DRG neuron following nerve injury. (**B**) Histone methylation regulates the neuron survival and axon degeneration in RGC or OPCs following nerve injury. (**C**) Histone methylation modulates the cell proliferation, axon guiding and myelin thickness of Schwann cell following nerve injury. (**D**–**H**) Histone methylation regulates the function of DRG and dorsal horn neurons in pain following nerve injury. DCA (Dorsal column axotomy); SNA (Sciatic nerve axotomy); TBI (Traumatic brain injury); RGC (Retinal ganglion cell); OPCs (Oligodendrocyte precursor cells); CCI-IoN (Chronic constriction injury of the infraorbital nerve); TG (Trigeminal ganglion).

In addition to DNA methylation, histone methylation also plays a crucial role in nerve injury, as histones are closely associated with DNA in the chromatin structure. Table 2 summarizes studies on histone methylation during nerve injury, including details on animal species, nerve injury models, experimental timelines, methylation detection methods, sample types and specific histone methylation type observed. Figure 2 illustrates how histone methylation regulates axon regeneration, neuron survival and degeneration, Schwann cell function and pain modulation following nerve injury.

### 4.1. Brain Trauma

In a rodent model of moderate controlled cortical impact (CCI) using postnatal day (PND)17 rats, the decreased histone H3 methylation was observed in the hippocampal CA3 region at 6 h post-injury and persisted for up to 3 days [157]. And another report indicated that diverse histone H3 methylation changes in the IGF promoter occur in different hippocampal regions in the CCI model. [158]. However, compared to DNA methylation, the role of histone methylation in brain trauma needs more research in the future to better understand its contributions.

### 4.2. Spinal Cord Injury

In spinal cord injury, quantitative chromatin immunoprecipitation (ChIP) assays revealed that H3K9ac, H3K9me2 and H3K27me3 were enriched on most promoters during dorsal column axotomy (DCA) and sciatic nerve axotomy (SNA). However, only H3K9ac and H3K9me2 were found to be differentially enriched on the promoters of axon regeneration genes, Growth associated protein 43 (*Gap43*)*, Galanin* and *Bdnf*. Among these, H3K9me2, a gene silencing-associated modification, was decreased at these promoters and negatively correlated with gene expression following SNA [159]. Moreover, emerging evidence demonstrates that inhibition of HDAC3, which catalyzes the deacetylation of H3K9ac, could overcome regenerative failure of sensory axons following spinal cord injury [160], and the effect of H3K9ac could explain the absence of methylation-derived effects.

The weak correlation between gene expression and histone methylation suggests that histone methylation may not directly regulate axon regeneration in the PNS or CNS but might be part of a broader, indirect regulatory mechanism.

### 4.3. Optic Nerve Injury

The elevated synthesis of H3K9me3, catalyzed by the histone methyltransferase EHMT2, critically contributes to RGCs loss and axonal degeneration following TBI [161]. This occurs through EHMT2-mediated direct suppression of nuclear factor E2-related factor 2 (*Nrf2*) transcriptional activity, which downregulates antioxidant enzyme mRNAs (e.g., superoxide dismutase (*Sod*) and *Catalase*) in both the RGCs and optic nerve, thereby exacerbating oxidative stress and neuronal damage. Notably, recent studies have highlighted a key role of EZH2 in axon regeneration following optic nerve injury. EZH2 enhances optic nerve regeneration and RGCs survival via histone methylation-dependent and -independent mechanisms, and *Slc6a13* acts downstream of EZH2 to control axon regeneration [162]. Conversely, the histone demethylase Kdm6a (an H3K27me3 demethylase) has been identified as an inhibitor of axon regeneration. Knockdown of Kdm6a promotes optic nerve regeneration and increases RGCs survival in both optic nerve crush (ONC) and NMDA-induced damage models [163]. It is suggested that increasing the levels of H3K9me3 and H3K27me3 can promote the regeneration of optic nerve axons and the survival of RGCs.

### 4.4. Sciatic Nerve Injury

Previous reports reviewed the histone methylation changes in H3K4me2/3, H3K9me2/3 and H3K27me2/3 following nerve injury or during regeneration [164,165,166,167,168]. In the context of peripheral axon regeneration, histone KMTs play a crucial role in DRG neurons. EZH2, a histone methyltransferase for H3K27me3, is upregulated in DRG during SNA in rats. Knocking down EZH2 inhibits axon regrowth in DRG neurons, and mice with EZH2 deficiency in DRG exhibit impaired sciatic nerve regeneration after injury [162,169]. Interestingly, interference with H3K27me3 demethylases such as Kdm6a or KDM6B promotes axon regeneration post-sciatic nerve injury [163]. These results demonstrate that H3K27me3 activation enhances the intrinsic regenerative capacity of DRG neurons following peripheral nerve injury, highlighting its potential as a therapeutic target for promoting axonal regeneration.

Emerging research has also examined the effect of histone methylation on Schwann cell function. The EED (embryonic ectoderm development)-containing polycomb repressive complex 2 (PRC2) catalyzes histone H3K27 methylation and is required for nerve repair following sciatic nerve injury. Mice with Schwann cell-specific deletion of EED show delayed neuronal regeneration and reduced expression of axon-guiding genes, such as semaphorin 4F (*Sema4F)* and ciliary neurotrophic factor (*Cntf)* [170], while specific deletion demethylases of H3K27me3 in the Schwann cell do not affect cell proliferation [25]. Moreover, the PRC2 complex acts as an epigenomic modulator of myelin thickness, a process linked to changes in Akt phosphorylation [171]. Thus, H3K27me3, by activating PRC2-induced axon-guiding genes, may be essential for Schwann cell function and sciatic nerve repair.

Histone modifications also play a significant role in neuropathic pain models. Peripheral nerve injury increases the activity of KMTs, including EHMT2, EZH2 and SUV39H1, in DRG neurons. EHMT2 primarily represses the transcription of K+ channels, facilitating the transition from acute to chronic pain after nerve injury [24]. Moreover, increased EHMT2 also contributes to the injury-induced suppression of opioid receptor mu 1 (*Oprm1*) in DRG neurons by blocking the access of cyclic AMP response element-binding protein to its promoter [172]. Similarly, EHMT2 regulates the downregulation of *Kcna2* and cannabinoid CB1 receptors in DRG neurons [173,174]. And increased SUV39H1 in neuronal nuclei of DRG and dorsal horn neurons, particularly small DRG neurons, contributes to allodynia and hyperalgesia by regulating mu-opioid receptor expression post-injury [175].

Meanwhile, in a rat model of partial sciatic nerve ligation, increased EZH2 and H3K27me3 expression in the spinal dorsal horn, particularly in microglia, was observed on days 3 and 10 post-injury. An EZH2 inhibitor reduced mechanical and thermal hyperalgesia in these rats [176]. Interestingly, calcitonin gene-related peptide (CGRP) is implicated in neuropathic pain by modulating EZH2, which induced H3K27m3 in microglial activation in the spinal dorsal horn after CCI [177]. Meanwhile, SNL also induces the upregulation of KDM6B, which facilitates the binding of STAT3 to the *Il6* promoter and enhances *Il6* expression in DRG and the dorsal horn, contributing to the maintenance of neuropathic pain [178]. Furthermore, H3K9me2 and H3K27me3 mediated downregulation of miR-32-5p in sensory neurons, affecting pain behaviors by targeting Cav3.2 channels [179]. In summary, EHMT2 and SUV39H1-mediated H3K9me3, along with EZH2-induced H3K27me3, may serve as upstream epigenetic regulators in neuropathic pain development following nerve injury. The upregulated expression of these KMTs and KDMs in DRG and spinal dorsal horn neurons appears to modulate multiple pain-related pathways. This epigenetic regulation potentially targets (1) ion channel genes (including potassium channels and Cav3.2 calcium channels), (2) pain-modulating receptor genes (such as *Oprm1*) and (3) inflammatory mediators (e.g., *Il6*). These coordinated molecular changes may collectively contribute to the pathogenesis of neuropathic pain through altered gene expression patterns.

Notably, the aforementioned studies were exclusively conducted using bulk tissue samples. However, epigenetic heterogeneity in histone methylation modifications may exist across distinct cell populations following peripheral nerve injury. Such cellular variability could potentially confound experimental interpretations, thus necessitating single-cell resolution analyses (scATAC-seq or scChIP-seq) to delineate cell type-specific epigenetic alterations.

Additionally, the differential expression of protein arginine methyltransferases (PRMTs) has been noted after nerve injury [180,181,182]. Spinal coactivator-associated arginine methyltransferase 1 (*Carm1*), which regulates histone arginine methylation, is downregulated after nerve injury. This reduction leads to decreased H3R17me2 levels at K+ channel promoters, resulting in epigenetic silencing of these channels and the development of neuropathic pain [183].

**Table 2:** List of articles on histone methylation in nerve injury and regeneration.

Animal	Injury Model	Timeline	Methods	Methylation Type	Sample	Ref.
Rat	SNL	0, 3 weeks	WB	H3K9me2, H3K27me3	DRG	[24]
Mice	SNT	0, 1 day	ChIP-Seq, ChIP-PCR	H3K27me3	Sciatic nerve	[26]
Rat	CCI	0, 6, 24, 72 h	IHC	H3K9me2, H3K4me2	Hippocampus	[157]
Rat	CCI	0, 3, 14 days	ChIP	H3K9me2/3, H3K27me3, H3K4me 3, H3K36me3	Hippocampus	[158]
Mice	DCA/SNA	0, 1, 3, 7 days	ChIP	H3K9me2, H3K27me3	DRG	[159]
Mice	CCI	0, 7 days	WB, IF	H3K9me2	RGC	[161]
Rat	SNL	0, 3, 10 days	WB	H3K27me3	Spinal dorsal horn	[176]
Rat	CCI	0, 1, 3, 5, 7, 10, 14 days	WB, IF	H3K27me3	Spinal dorsal cord	[177]
Rat	SNL	0, 1, 3, 7, 10, 14 days	WB	H3K27me3	DRG, dorsal horn	[178]
Rat	SNL	0, 7 days	WB, IF	H3R17me2	Spinal dorsal horn	[183]

Note: SNL (Spinal nerve ligation); SNT (Spinal nerve transection); CCI (Controlled cortical impact); DCA (Dorsal column axotomy); SNA (Sciatic nerve axotomy); IHC (Immunohistochemical staining); ChIP (Chromatin immunoprecipitation); WB (Western blot); IF (Immunofluorescence staining); ChIP-Seq (Chromatin immunoprecipitation-Gnen Sequence); ChIP-PCR (Chromatin immunoprecipitation-Polymerase Chain Reaction); RGC (Retinal ganglion cell).

## 5. The Role of RNA Methylation in Nerve Injury

**Figure 3:**
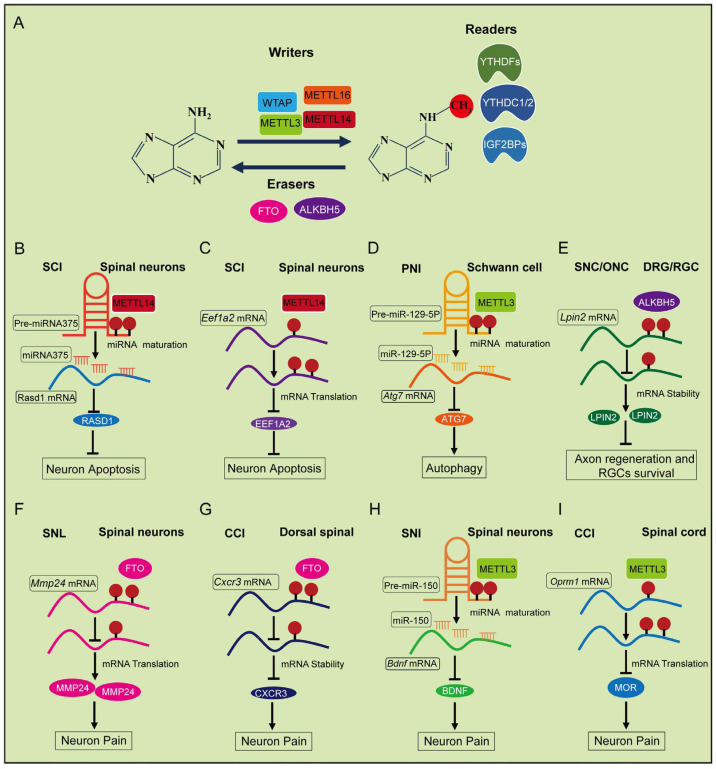
The mechanism of RNA m^6^A methylation in neuronal cells following nerve injury. (**A**) The schematic diagram of RNA m^6^A methylation modulated by Writers (methyltransferases, e.g., METTL3, METTL14, WTAP and METTL16), Erasers (demethyltransferases, e.g., FTO and ALKBH5), and Readers (binding proteins for RNA m^6^A, e.g., YTHDFs, YTHDC1/2 and IGF2BPs) proteins. (**B**,**C**) RNA m^6^A methylation regulates the apoptosis of spinal neurons following spinal cord injury. (**D**,**E**) RNA m^6^A methylation regulates Schwann cell autophagy and axon regeneration in RGC and DRG neurons following sciatic nerve injury. (**F**–**I**) RNA m^6^A methylation regulates the function of spinal neurons in pain following nerve injury. SCI (Spinal cord injury); SNC (Sciatic nerve crush); ONC (Optic nerve crush); PNI (Peripheral nerve injury).

RNA methylation is essential for regulating RNA synthesis and metabolism. Emerging research has specifically demonstrated the importance of m^6^A methylation in nerve injury, whereas the roles of m^1^A and m^5^C modifications in RNA have resulted in new directions for future investigations. Table 3 provides a comprehensive summary of studies on RNA m^6^A methylation in nerve injury, including animal species, nerve injury models, experimental timelines, methylation detection methods and examined sample types. Moreover, m^6^A-mediated regulation has been shown to impact key processes following nerve injury, including axon regeneration, neuron survival, pain modulation and nerve degeneration (Figure 3).

### 5.1. Brain Trauma

There is growing evidence that RNA methylation, particularly m^6^A modification, plays a critical role in various physiological and pathological processes. Currently, only two studies have explored the function of m^6^A modifications and their associated enzymes in brain trauma. One study reported that m^6^A modifications were differentially altered, with an increase in METTL3 expression observed in the hippocampus during the early stages of TBI [184]. Conversely, another study found that the expressions of METTL14 and FTO were significantly downregulated in the cerebral cortex of rats following TBI. Notably, functional FTO was shown to be essential for repairing neurological damage caused by TBI. However, inhibition of FTO using the inhibitor FB23-2 did not affect the spatial learning and memory abilities of TBI-affected rats [185]. In addition to m^6^A modifications, a recent report indicated profound changes in the m^5^C methylation profiles of lncRNAs in the rat brain following TBI [186]. These findings highlight the potential significance of m^6^A and m^5^C modifications and their regulatory enzymes in the pathophysiology of brain trauma, suggesting new avenues for therapeutic exploration.

### 5.2. Spinal Cord Injury

Recent studies have highlighted the role of m^6^A RNA methylation in SCI [187]. During the acute phase of SCI, six m^6^A regulators were specifically expressed in leukocytes from peripheral blood. Among these, the expressions of *Fto* (an eraser) and RNA binding motif protein X-linked (*Rbmx*) (a reader) were significantly downregulated [188]. Moreover, alterations in m^6^A RNA methylation profiles following spinal cord injury (SCI) have been reported in both rats and mice. In rats, changes in m^6^A levels in both mRNA and lncRNA within the spinal cord were detected after spinal cord contusion [189]. In mice, METTL3 expression was upregulated in GFAP-positive astrocytes and nestin-positive neural stem cells after SCI [190]. Additionally, METTL14 expression and m^6^A modifications were detected in spinal cord tissues. Inhibiting *Mettl14* promoted motor function recovery by reducing neuronal apoptosis, partly through the suppression of miR-375 maturation [191], Furthermore, silencing *Mettl14* attenuated neuronal apoptosis and improved outcomes after SCI by reducing m^6^A modification of eukaryotic translation elongation factor 1 alpha 2 (*Eef1a2*) [192]. A recent report also showed that after spared nerve injury, 55 differentially expressed and m^6^A-methylated genes were identified in the spinal cord. Additionally, the expressions of m^6^A reader enzymes *Ythdf2* and *Ythdf3* were upregulated in the lumbar spinal cord [193]. In addition to m^6^A, differentially expressed genes associated with RNA m^5^C modification have been detected in rat SCI models, suggesting that RNA m^5^C modification may be involved in the spinal cord injury process [194]. Despite these findings, the functional implications of these m^6^A modifications in SCI also need further validation, and whether manipulating m^6^A-modified genes can enhance axonal regeneration or improve functional recovery following SCI warrants further investigation.

### 5.3. Optic Nerve Injury

In the context of optic nerve injury, knocking down the m^6^A “writer” protein complex component *Mettl14* impaired axon regeneration and reduced the survival rate of RGCs in *Pten* conditional knockout (cKO) mice post-injury. However, the survival rate of alpha-RGCs (αRGCs) remained unaffected [27]. Previous studies have demonstrated that αRGCs are preferentially resilient to injury and are primarily responsible for axon regeneration following *Pten* deletion [195]. These findings suggest that *Mettl14* may play a critical role in the survival or axonal regeneration of other types of RGC neurons, beyond αRGCs, although the precise phenotype of these neurons requires further investigation. Additionally, recent research has identified the m^6^A demethylase ALKBH5 as an inhibitor of axon regeneration. Interfering with *Alkbh5* expression in RGCs using adeno-associated virus (AAV) was shown to promote both optic nerve regeneration and RGCs survival [29]. These results highlight the importance of m^6^A modifications in regulating neuronal survival and axon regeneration after optic nerve injury, offering potential targets for therapeutic intervention.

### 5.4. Sciatic Nerve Injury

Recent studies have reported differential m^6^A RNA methylation patterns in nerve injury and regeneration [196,197]. Increased m^6^A modifications were observed in DRG neurons following sciatic nerve injury. Conditional knockout of the m^6^A methyltransferase complex *Mettl14* or the reader protein *Ythdf1* impaired axon regeneration and delayed functional recovery by affecting protein translation [27]. Additionally, differential expression of m^6^A-related genes and alternative m^6^A methylation levels were detected in the sciatic nerve after injury [197]. Knocking down the m^6^A demethylase ALKBH5 promoted axonal regeneration. ALKBH5 was shown to increase the stability of lipin 2 (*Lpin2)* mRNA via an m^6^A motif in its 3′ UTR in a demethylase-dependent manner, thereby limiting regenerative growth-associated lipid metabolism in DRG neurons [29]. Furthermore, METTL3-mediated maturation of miR-192-5p, which targets autophagy-related 7 (*Atg7)*, was found to prevent Schwann cell autophagy during peripheral nerve injury [198].

In the neuropathic pain (NP) model, m^6^A RNA methylation plays a crucial role in the development of pain and associated behavioral changes. In the spinal cord, spinal nerve ligation enhances the binding of FTO to matrix metallopeptidase 24 (*Mmp24)* mRNA, promoting its translation and contributing to NP genesis [199]. FTO also increases the mRNA stability of *Cxcr3*, further exacerbating NP [200]. METTL3 accelerates the maturation of miR-150 by mediating m^6^A methylation of pri-miR-150. This process involves cooperation with the m^6^A reader YTHDF2 and directly targets *Bdnf* mRNA, a key factor in pain modulation [201]. Recent studies have shown that METTL3 suppresses the expression of *Mor* by regulating the methylation of *Oprm1* mRNA, influencing nociceptive pathways in NP [202]. These findings highlight the importance of RNA m^6^A methylation in regulating neuropathic pain, particularly through its effects on axonal regeneration, behavioral responses and functional recovery in the nervous system. While m^6^A modifications are clearly implicated in both NP and SNI, the precise roles and mechanisms of proteins involved in these processes remain to be fully elucidated. This underscores the need for further research to explore how m^6^A modifications might be targeted therapeutically for nerve repair and pain management.

**Table 3:** Altered RNA m^6^A modification during nerve injury and regeneration.

Animal	Injury Model	TimeLine	Methods	Sample	Ref.
Mice	Sciatic nerve crush	0, 1 day	m^6^A-SMART-seq, m^6^A-CLIP-SMART-seq	L4-L5 DRG	[27]
Rat	Controlled cortical impact	0, 1 day	MeRIP-seq	Cerebral cortex	[185]
Rat	T10 spinal cord contusion	0, 4 weeks	MeRIP-Seq	Spinal cord	[189]
Rat	Sciatic nerve injury	0, 7 days	MeRIP-seq	Sciatic nerve	[197]
Rat	Middle cerebral artery occlusion and reperfusion model	0, 1 day	MeRIP-seq	Brain cortex	[203]

Note: m^6^A-SMART-seq (Methylated RNA immunoprecipitation and switching mechanism at 5′end of the RNA transcript sequencing); m^6^A-CLIP-SMART-seq (Methylated RNA crosslinking-immunoprecipitation and switching mechanism at 5′end of the RNA transcript sequencing); MeRIP-seq (Methylated RNA immunoprecipitation sequencing).

## 6. Future Perspectives

This review comprehensively described the predominant effects of methylation modifications on DNAs, histones and RNAs in regulating gene expression during nerve injury and regeneration. Both CNS and PNS injuries induce changes in the methylation profiles of DNAs, histones and RNAs. However, there are differences in the methylation patterns observed in the same nerve in different injury models. One possible explanation for these discrepancies is the use of different detection methods or tools, as well as the varying response degree of methylation modifications across different injury models. Moreover, current methylation research predominantly relies on bulk tissue analyses, which inherently mask the epigenetic heterogeneity among distinct neural cell populations exhibiting divergent responses to injury. This methodological limitation leads to dilution of cell type-specific methylation signatures and instability in epigenetic measurements. Implementing single-cell resolution epigenomic profiling represents a compelling strategy to precisely map cell class-specific methylation alterations during neural repair.

Furthermore, most studies focus on methylation changes in a single dimension, such as DNA methylation, histone methylation or RNA methylation. However, the orchestration of gene expression by methylation modifications in all these dimensions or their interactions with other epigenetic modulators should be fully considered in studies of nerve injury and regeneration. Increasing reports suggest that there is significant crosstalk in the methylation modification effects between DNAs, histones and RNAs [204,205]. Notably, advancements have been made by manipulating methylation modifications in DNAs, histones or RNAs through specific inhibitors in nerve injury-induced pain, offering valuable insights into the effects of methylation modifications on nerve injury and regeneration [206,207]. These results mark a significant step forward in clinical applications.

This review summarized recent advances in methylation modifications during nerve injury and regeneration. However, the mechanisms by which methylation regulates gene expression across different dimensions remain incompletely understood. For instance, most studies have concentrated on mRNA expression patterns regulated by RNA methylation during nerve injury [27,208], with growing evidence indicating that non-coding RNAs are also regulated by RNA methylation modifications [209]. Our previous findings demonstrated that a large number of non-coding RNA molecules are involved in axon regeneration [210,211], but it remains unclear whether these molecules are regulated by methylation. Additionally, the development of new methods or tools for precisely identifying methylation changes at a single nucleotide resolution during nerve injury and regeneration would be immensely helpful.

Several important considerations should guide future research. First, attention should be paid to the upstream signals or events that govern methylation modifications, including intrinsic factors or extrinsic signals that regulate specific methylation sites. Recent reports have primarily focused on downstream target genes during nerve injury and regeneration, but emerging data suggest that methylation modifications act as dynamic markers controlling gene expression in response to external stimuli and signals. Second, understanding the diverse functions of dynamic methylation modifications in DNA, histones and RNA at various stages of nerve injury and regeneration, particularly during the initiation and extension phases of injury and regeneration, remains a critical area of research.

Third, there is a need for the development of specific inhibitors or activators for proteins involved in methylation modifications. While some inhibitors and activators have been used in these processes, most are broad-spectrum and non-specific, potentially explaining some of the variable results observed. Advancing the synthesis of newly optimized, targeted compounds through artificial intelligence (AI) technology will accelerate the application of DNA methylation modifications in treating and recovering from nerve injury.

Lastly, the crosstalk between methylation and other modifications needs to be explored. Understanding whether dynamic changes in methylation impact other modifications involved in gene expression modulation during nerve injury and axon regeneration and vice versa will provide critical insights into how these processes are coordinated. Further advances in the understanding of methylation modifications will be driven by continued research and exploration in the context of nerve injury and regeneration.

In conclusion, emerging evidence underscores that dynamic methylation modifications in DNA, histones and RNAs serve as critical epigenetic regulators of gene expression during nerve injury and regeneration. The application of advanced detection methods, such as single-cell epigenomic profiling, is critical for mapping the intricate regulation of methylation in multi-layered gene expression networks following nerve injury. Moreover, pharmacological screening targeting methylation regulators will significantly accelerate translational progress in nerve repair.

## Data Availability

All relevant data are within the paper.
